# An electrochemical proximity assay (ECPA) for antibody detection incorporating flexible spacers for improved performance

**DOI:** 10.1007/s00216-024-05546-9

**Published:** 2024-10-05

**Authors:** Amanda S. N. Kurian, Mainul Islam Mazumder, Asanka Gurukandure, Christopher J. Easley

**Affiliations:** Department of Chemistry and Biochemistry, Auburn University, Auburn, AL 36849 USA

**Keywords:** Bioanalytical methods, Biosensors, Clinical/biomedical analysis, Electroanalytical methods, Electrochemical sensors/mass sensitive sensors, Immunoassays/ELISA

## Abstract

A clever approach for biosensing is to leverage the concept of the proximity effect, where analyte binding to probes can be coupled to a second, controlled binding event such as short DNA strands. This analyte-dependent effect has been exploited in various sensors with optical or electrochemical readouts. Electrochemical proximity assays (ECPA) are more amenable to miniaturization and adaptation to the point-of-care, yet ECPA has been generally targeted toward protein sensing with antibody-oligonucleotide probes. Antibodies themselves are also important as biomarkers, since they are produced in bodily fluids in response to various diseases or infections, often in low amounts. In this work, by using antigen-DNA conjugates, we targeted an ECPA method for antibody sensing and showed that the assay performance can be greatly enhanced using flexible spacers in the DNA conjugates. After adding flexible polyethylene glycol (PEG) spacers at two distinct positions, the spacers ultimately increased the antibody-dependent current by a factor of 4.0 without significant background increases, similar to our recent work using thermofluorimetric analysis (TFA). The optimized ECPA was applied to anti-digoxigenin antibody quantification at concentrations ranging over two orders of magnitude, from the limit of detection of 300 pM up to 50 nM. The assay was functional in 90% human serum, where increased ionic strength was used to counteract double-layer repulsion effects at the electrode. This flexible-probe ECPA methodology should be useful for sensing other antibodies in the future with high sensitivity, and the mechanism for signal improvement with probe flexibility may be applicable to other DNA-based electrochemical sensor platforms.

**Graphical Abstract:**

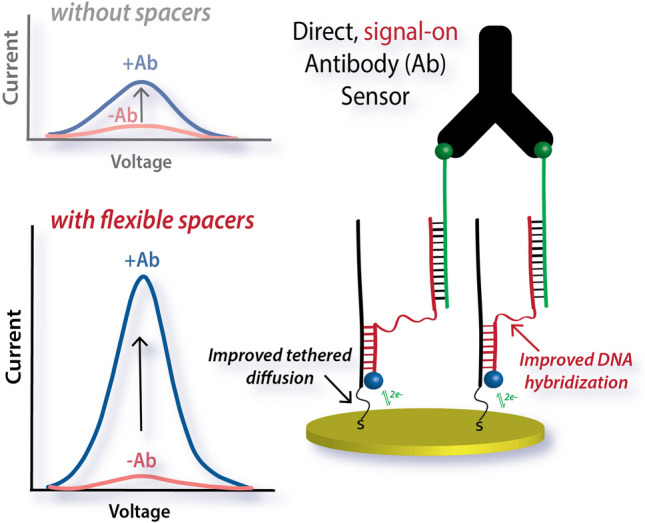

**Supplementary Information:**

The online version contains supplementary material available at 10.1007/s00216-024-05546-9.

## Introduction

Biomarkers such as DNA, RNA, enzymes, antibodies, and antigens can be measured [1–3] to predict, diagnose, and monitor diseases in clinical practice [3]. Many biomarkers are found in very low concentrations in blood and other bodily fluids [4], necessitating highly sensitive measurement. Rapid biomarker detection can facilitate point-of-care diagnosis. Thus far, a variety of methods such as the absorbance-based enzyme-linked immunosorbent assay (ELISA) [5], surface plasmon resonance (SPR)-based immunoassays [6], and fluorescence turn-on nanoprobes [7] have been applied for biomarker detection. Among these methods, ELISA and its successors Alpha-LISA [8] and SiMoA [9] are considered as gold standard methods because of their high sensitivity and reliability. However, Alpha-LISA requires specialized instrumentation, and SiMoA and ELISA are time-consuming and labor-intensive. There is clearly a need for a fast and cost-effective alternative.

Advanced biomarker sensors have been developed to overcome the limitations of ELISA. The proximity ligation assay (PLA), for example, combines the specificity of ELISA and sensitivity of PCR [10–12]. PLA exploits two proximity probes (antibody or aptamer conjugated to ssDNA) that bind to the same antigen. When the PLA probes are in proximity, the two ssDNA can undergo hybridization with two other sequence-specific oligonucleotides, and PCR amplification with fluorophore-tagged oligos gives high detection sensitivity [13]. Some reports have even used PLA to visualize the presence of analytes with a fluorescence microscope [14, 15]. Low sample consumption, cost-effective instrumentation, and simpler experimental workflow make PLA more efficient than ELISA [16], and the method’s sensitivity is 3–4 orders of magnitude higher than ELISA [17]. Several other biosensors have been reported to utilize the proximity effect due to its high sensitivity. ADAP (antibody detection by agglutination-PCR) methods have used the proximity effect to monitor antibodies in blood after immunization [18, 19] and multiplex analysis of islet autoantibodies [20, 21]. Although PLA techniques are highly sensitive, the need for specific benchtop instrumentation and prolonged analysis (a few hours) hinders their applicability in point-of-care diagnostics.

Biomarker concentration can be measured with simpler instrumentation as a function of current or impedance using electroanalytical methods. DNA-driven electrochemical biosensors leverage the immobilization of oligonucleotide strands tagged with a redox reporter such as methylene blue (MB) [22, 23], or ferrocene [24, 25] on a gold surface using a self-assembled monolayer [26–28]. When a potential is applied, subtraction of non-faradaic current can yield analyte-dependent faradaic current [29]. Major advantages of electrochemical biosensors are easy-to-operate instrumentation, cost-effective sensing elements, and availability of various efficient and rapid transduction methods [30–32]; and the high sensitivity, good specificity, and compatibility with miniaturization have made electrochemical systems indispensable for point-of-care diagnostics [33–35]. Varied electrochemical sensors, such as electrochemical aptamer-based sensors (E-AB) [33, 36–39], E-DNA scaffolds [35, 40–42], a molecular pendulum [24], and a bowtie-shaped DNA nanostructure for immunosensing [43–45], are promising for point-of-care measurements [46]. Previously, our group reported the electrochemical proximity assay (ECPA) capable of detecting proteins as low as the fM range [47]. ECPA utilizes the proximity binding concept to move an electroactive species (MB) closer to the gold electrode in an analyte-dependent way [47–50]. Target flexibility, reusability, high sensitivity, and wide dynamic range are notable strengths of the ECPA method [48, 50]. In this manuscript, most of these attributes are leveraged, although the reusability feature was not yet explored due to the complexity of adding enzymatic DNA cleavage (uracil DNA excision) into the experimental system alongside multi-site flexibility changes.

Immunity against antigens and autoimmune disease management could greatly benefit from antibody sensing in patients’ blood. Hence, the detection and quantification of antibodies in human blood are critical [51, 52]. Here, we present a novel iteration of ECPA, where the arrangement of the molecular complexes extends from our recently reported thermofluorimetric analysis (TFA) [53].

In this ECPA variation, antibody binding brings a redox moiety (MB) closer to the surface, and resultant square-wave voltammetric (SWV) current is proportional to antibody concentration. Similar to the TFA work [53], herein, we emphasize the importance of DNA strand flexibility in assay performance. Probe flexibility was achieved with polyethylene glycol (PEG) linkers [41, 54–56] added to the DNA strands. Results demonstrated clearly that including PEG improved analyte-dependent currents. Interestingly, the placement of the spacers provided two distinct enhancements: improved DNA hybridization yield and increased SWV current. Ultimately, the limit of detection (LOD) was as low as 300 pM for anti-digoxigenin antibodies—without using enzymatic signal amplification—and the system functioned well in both buffer and human serum. Compared to TFA, this electrochemical version of the antibody sensor thus exhibited better sensitivity and reduced assay time. Comparisons to prior ECPA iterations are less trivial, since this manuscript is the first to report an ECPA antibody sensor, but the internal control in this work shows that a 4.0-fold improvement in sensitivity is achievable with enhanced probe flexibility.

## Materials and methods

### Reagents

Customized DNA strands—with and without digoxigenin (dig) modifications and PEG spacers—were purchased from Integrated DNA Technologies (IDT) (Coralville, IA), Biosynthesis (Lewisville, TX), and Biosearch Technologies (Novato, CA). These strands were purified by HPLC, and purity and yield were verified by mass spectrometry. The DNA strand sequences are given in Table S-1. Sodium chloride was obtained from BDH. 4-(2-Hydroxyethyl)-1-piperazineethanesulfonic acid (HEPES) was from Alfar Aesar. Bovine serum albumin (BSA) was purchased from OmniPur. Tris-(2-carboxyethyl) phosphine hydrochloride (TCEP), mercaptohexanol (MCH), chromium etchant, and gold etchant were purchased from Sigma-Aldrich (St. Louis, MO). AZ 40XT positive photoresist and AZ 300 MIF developer were obtained from Microchemicals. Polydinethylsiloxane (PDMS) was from Dow Corning, and dimethyl sulfoxide (DMSO) was purchased from Anachemia. Gold-on-glass (GoG) slides with dimensions of 25.4 mm × 330.2 mm × 1.1 mm (Cr adhesion layer ~ 5 nm and Au adhesion layer ~ 100 nm) were purchased from Deposition Research Labs, Inc. (St. Charles, MO). Anti-digoxigenin antibody (mouse monoclonal) was purchased from Roche and human serum from BioIVT. Necessary buffers and solutions were prepared in deionized, ultra-filtered water (Thermo Fisher Scientific). Electrochemical cell preparation, electrode fabrication, and illustrations are given in supplementary information (Fig. S-1).

### SDNA monolayer assembly

Thiolated DNA (thio-DNA) (20 µM, 1.0 µL) was reduced with TCEP (10 mM, 3.0 µL) by incubating for 1 h in dark. This solution was diluted with immobilization buffer (10 mM HEPES, 0.5 M or 1.0 M NaCl) to a final concentration of thio-DNA at 125 nM. 100 µL of this solution was added to the electrochemical (EC) cell with a gold-on-glass (GoG) working electrode and was incubated for 1 h in the dark. Next, 100 µL of 3 mM MCH was added to the electrode and incubated further for another 1 h (in dark), followed by a 30-min incubation with assay buffer (10 mM HEPES, 0.5 M or 1.0 M NaCl, and 0.1% BSA). Monolayer-assembled electrodes were stored in the refrigerator until EC measurements were performed.

### Anti-digoxigenin antibody detection in buffer

Initially, digoxigenin-tagged DNA (dig-DNA) and methylene blue DNA (MB-DNA) were mixed and incubated at room temperature for 1 h. This step can be accomplished as a pre-incubation and should not be included in the assay workflow time. Next, anti-digoxigenin antibodies (anti-dig) were added to the DNA mixture and were allowed to incubate at room temperature for 30 min. 15 µL of this mixture was added to the GoG working electrode and was incubated for 40 min at room temperature. During this time, the MB-DNA was shown to hybridize with thio-DNA, nearing equilibrium. The 40-min timing was chosen as a compromise between experimental analysis time and time to reach complete equilibrium, and this time was held consistent throughout the measurements. The background (BG) included assay buffer instead of the antibody. Final concentrations of MB-DNA were 50 nM, anti-dig was 25 nM, and thio-DNA was 125 nM. After the final incubation step, solution was removed from the electrode, followed by the addition of 100 µL of assay buffer, and SWV scans were performed. To generate a calibration curve, a series of anti-dig solutions ranging from 0 to 100 nM were electrochemically measured as described above. Final concentrations of dig-DNA and MB-DNA were 50 nM, whereas thio-DNA was 125 nM.

### Anti-digoxigenin detection in human serum

Electrochemical measurements were performed to detect anti-dig antibodies (20 nM) in 90% human serum using incubation steps as mentioned previously. For one of the serum samples, 4.0 M NaCl was spiked to elevate the salt concentration in serum to approximately 0.45 M. To generate a BG for serum experiments, serum of equal volume to that of spiked antibody was added. After the 40-min incubation of 15 µL serum sample on the electrode, the solution was removed, followed by addition of 100 µL of assay buffer and SWV measurement.

### Electrochemical measurements

All measurements were performed using the Gamry 600 reference potentiostat. When the gold-on-glass (GoG) electrode was ready for measurement, it was connected to create a 3-electrode cell, with a silver/silver chloride reference electrode (3 M KCl) (BASi) and a platinum counter electrode (CH Instruments). Square-wave voltammetric (SWV) measurements were performed from − 0.45 to 0.00 V versus reference electrode, with a step size of 2 mV and pulse width of 50 mV at a frequency of 464 Hz. In some experiments, a range of SWV frequencies were scanned (3–1000 Hz) using the same step size and pulse width. Unless noted otherwise, all measurements were done in triplicate, using three separately fabricated sensors.

### Data analysis

Data processing was done using customized MATLAB codes and Microsoft Excel. First, the.DTA files obtained from the Gamry 600 were converted to.txt files using a Windows batch file. The necessary current versus voltage data was then obtained from.txt files by running customized MATLAB codes, and the baseline corrected current versus potential data was exported to Microsoft Excel for further processing, such as obtaining peak currents and percentage changes in signal. Details of data analysis and related graphical demonstrations are given in supporting information (Figs. S-2 and S-3).

## Results and discussion

### System design and sensing principle

Recently, our group developed an optical assay to quantify anti-dig at an LOD of 7 nM in buffer using TFA, and in this work, we demonstrated that polyethylene glycol (PEG) spacers within DNA strands aid efficient hybridization [53]. While this assay was functional in buffer and serum, several weaknesses exist with respect to clinical applications, namely the nanomolar LOD and the need for optical systems. Earlier, our group introduced electrochemical protein assays for insulin and thrombin [47, 50] which were more sensitive and more amenable to point-of-care, clinical settings. However, this ECPA has not yet been developed for antibody sensing. Here, we have adapted the DNA-driven complex formation used in the TFA antibody assay [53] to an electrochemical version, again leveraging the proximity effect. By borrowing the concepts on probe flexibility learned in optical methods, this novel antibody ECPA is optimized herein to achieve an LOD of 300 pM and functionality in human serum.

**Fig. 1:**
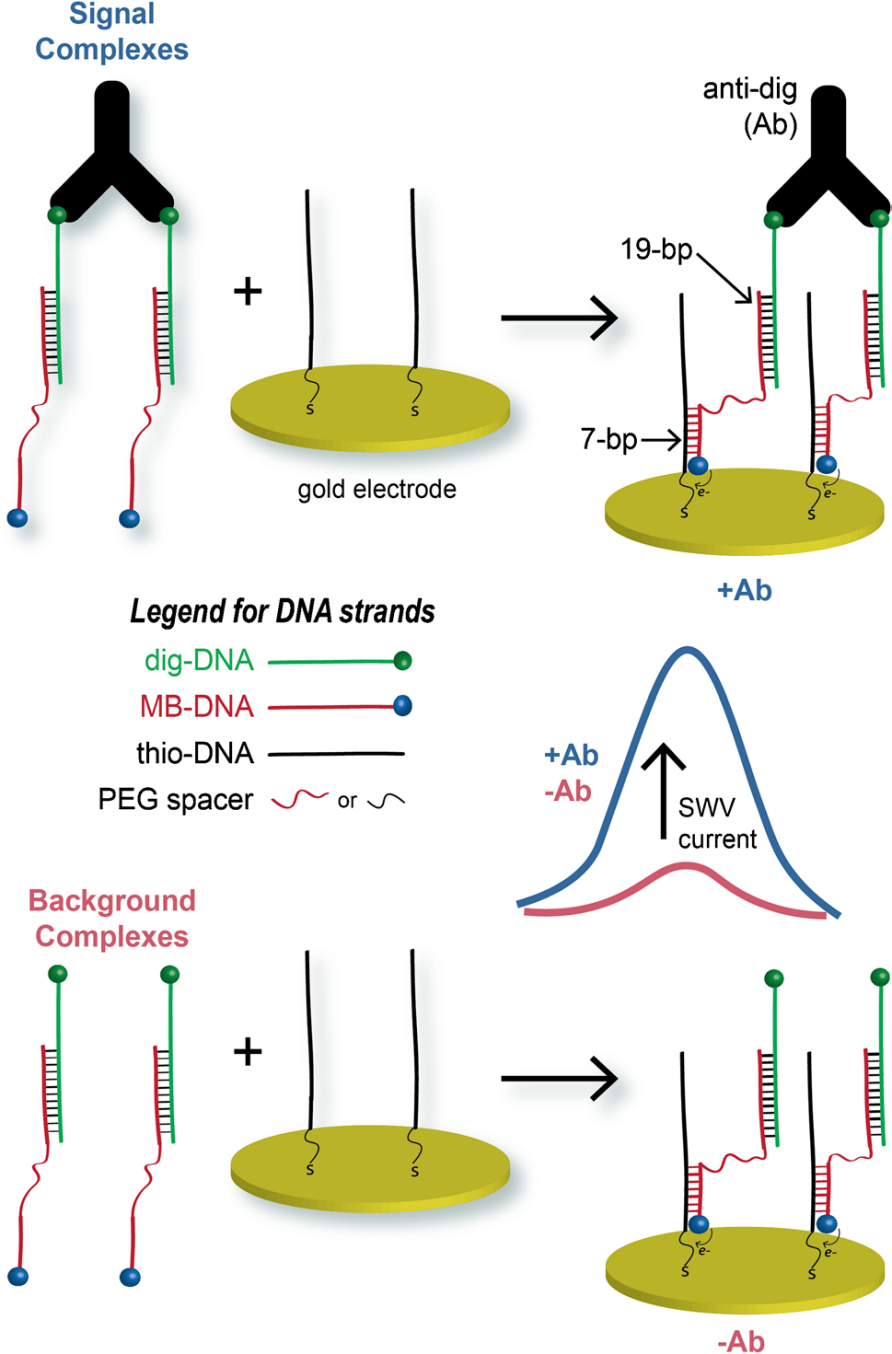
Sensing principle of ECPA adapted for antibody detection. More MB-DNA approaches the electrode surface in the presence of antibody (+ Ab; blue data) than in its absence (− Ab; red data) due to the proximity effect

Specifically, this antibody ECPA system design is a surface-based proximity assay for antibody quantification. It could be argued that the assay is an avidity-driven surface assay, yet because it is designed based on proximity assays and provides equivalent response curves, we chose to maintain the “proximity” in ECPA. The assay leverages three DNA strands: a thiol-modified DNA (thio-DNA) for electrode immobilization, an antigen-DNA conjugate (dig-DNA) for antibody binding, and a methylene blue-labeled DNA (MB-DNA) for signal generation. As illustrated in Fig. 1, each dig-DNA is hybridized to a single MB-DNA through 19 base pairs. Target antibody (Ab) addition induces the spontaneous binding of antigen-DNA conjugates (dig-DNA), promoting increased stability of hybridization of the two MB-DNA strands (only 7 bp each) to the surface thio-DNA—an effect referred to as the proximity effect. The close proximity of the two MB-DNA strands creates a bivalent binder that can much more stably associate with multiple thio-DNA strands on the electrode, compared to individual 7-bp segments. This effect is an entropic enhancement akin to the formation of a DNA hair hairpin loop with a short dsDNA segment, and in this assay format, it positions two MB molecules close to the electrode surface to allow efficient electron transfer at the electrode surface. Therefore, one Ab binding event results in signal from two MB molecules.

### Positional effects of spacers on assay performance

To evaluate the importance of probe flexibility, we used anti-digoxigenin antibody (anti-dig) as the target and digoxigenin-DNA conjugates (dig-DNA) as the antigens. For comparison, we first used two types of MB-DNA strands and two types of thio-DNA strands, either including flexible linkers or not.

**Fig. 2:**
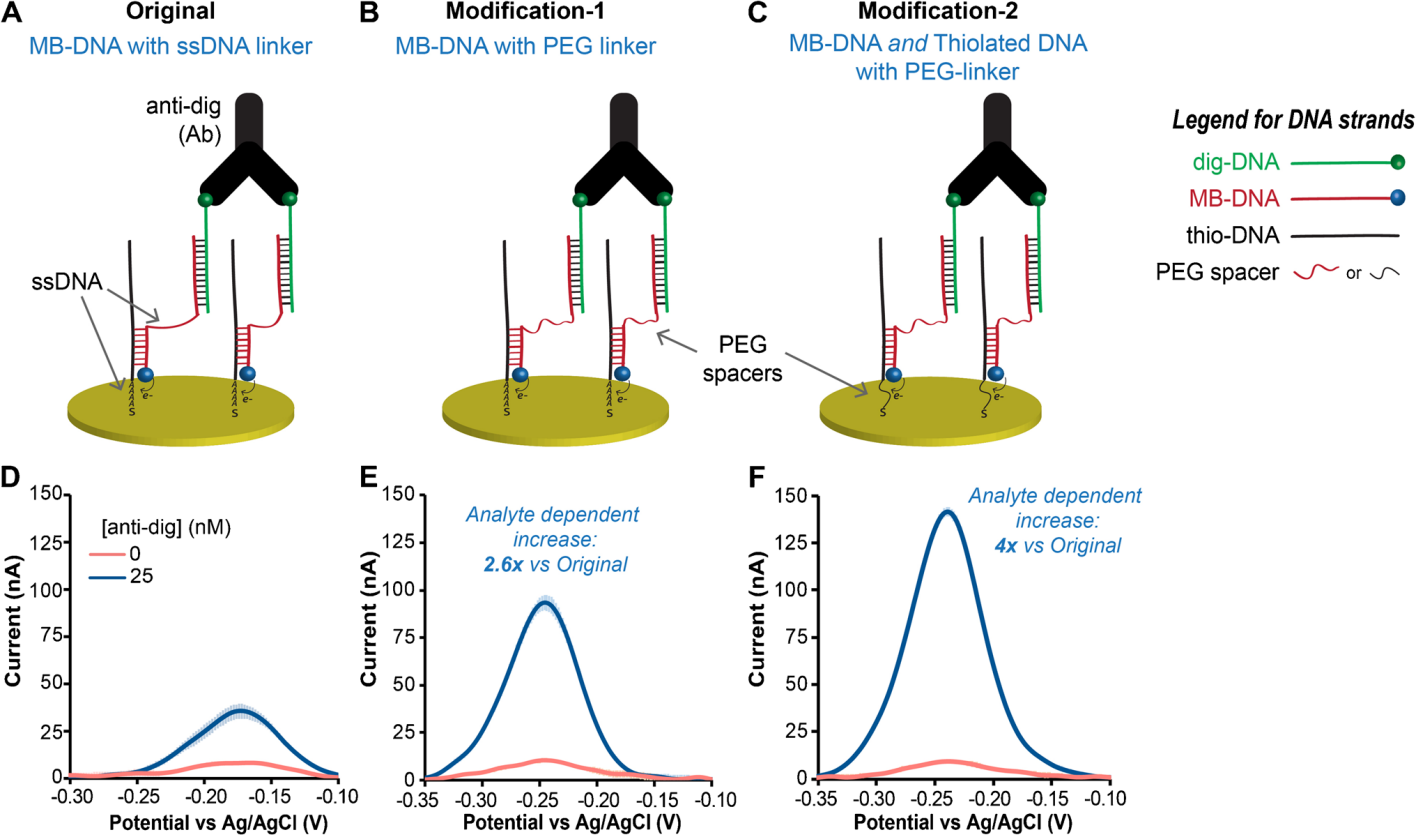
Effects of probe flexibility on the performance of antibody ECPA. **A** The original design with ssDNA linkers or spacers. **B** The first modification where a PEG linker of similar length was added to the MB-DNA strand. **C** Second modification where the ssDNA spacer in the thio-DNA was replaced by a PEG linker of similar length. Square-wave voltammograms of the original design (**D**), modification-1 (**E**), and modification-2 (**F**) either with antibody addition (blue) or with no analyte (pink) showed enhancements given from both modifications, up to a final fourfold improvement in current. Error bars represent the standard deviations of measurements from three separate electrodes, and SWV peak currents were measured at 464 Hz

One strand type includes a less flexible, ssDNA linker (MB-DNA) separating the 20-bp and 7-bp binding regions (Fig. 2A), and this was termed the **original** design. As mentioned previously, addition of anti-dig will promote MB-DNA to bind more strongly to thio-DNA on the gold surface via the proximity effect. The generated SWV current should be proportional to the concentration of target antibody, and the 7-bp connection length to the surface was selected based on several prior studies by our group [13, 47, 48, 50, 53] and others [54, 56]. In Fig. 2, we compared signals after addition of 25 nM of anti-dig (signal) to a background (BG) which did not include the target antibody. In the **original** design, target-induced hybridization resulted in a higher peak current compared to the BG, as shown in Fig. 2D.

To evaluate the effect of probe flexibility on MB-DNA strands, the next system design included DNA modified with polyethylene glycol linkers (PEG-MB-DNA) of similar length as the ssDNA, which was termed **modification-1** of the original design (Fig. 2B). With each PEG spacer representing about 2.4 nm [56] and a ssDNA length of 0.68 nm per nt [57], the PEG linker in this work represents about 9.6 nm, equivalent to about 14 nt of ssDNA length; this linker replaced 11 nt of the ssDNA spacer from the **original** design (see Table S-1). With this system, addition of 25 nM anti-dig gave a large enhancement in the SWV current (2.6-fold) (Fig. 2E) compared to the **original** design without a significant change of the BG current. Considering our recent work using TFA in an analogous assay format [53], it is likely that the added flexibility of the PEG-MB-DNA strands caused an enhancement of the proximity effect and further stabilized the DNA hybridization near the electrode surface. Indeed, the persistence length, a measure of polymer rigidity or flexibility, is ~ 50 nm for the fairly rigid dsDNA [58], ~ 2 nm for ssDNA [59], and only ~ 0.4 nm for the highly flexible PEG [60]. While ssDNA is certainly more flexible than dsDNA, the PEG spacers have a ~ fivefold lower persistence length, providing even more flexibility in our system. Thus, **modification-1** presumably resulted in the recruitment of more antibody-antigen-DNA complexes to the surface over time, giving more current and an improved anti-dig sensor.

Flexible spacers were then added into the thio-DNA strands, giving the **modification-2** system shown in Fig. 2C. It should first be noted that the ssDNA spacer (4-dA nucleotides) in the **original design** was included based on our previous work which showed that detrimental electric double-layer effects could be avoided by including a spacer between the electrode and a dsDNA hybridization site [61]. Thus, the spacer is a necessary part of the design. Here, we hypothesized that replacing the 4 dA segment in the thio-DNA with a PEG spacer of similar length would enhance the SWV current by increasing the rate of tethered diffusion. Indeed, with the **modification-2** system, addition of 25 nM anti-dig gave an even larger enhancement in the SWV current (4.0-fold) (Fig. 2F) compared to the **original** design, again without a significant change of the BG current.

### Mechanisms of SWV current enhancement

It is clear from data in Fig. 2 that adding flexible linkers into both the MB-DNA and thio-DNA strands gave drastic improvements in the SWV current. While we posed conjectures on the mechanistic reasoning behind these enhancements, further study was warranted. In this section, we show that a detailed study on SWV frequency dependence can reveal mechanistic information about the flexible linkers. First, using the blank (0 nM Ab) measurements with each system, it was determined that the surface density of hybridized MB-DNA strands was essentially unaffected by the addition of PEG spacers. For the **original** design, **modification-1**, and **modification-2**, the MB-DNA surface densities were 1.14, 1.45, and 1.30 fmol cm^−2^ (see Supporting Information, p. S-4). Since these values were similar and showed no obvious trends, we assumed that the flexible spacers did not have an appreciable effect on the surface packing density of the thiol-DNA strands.

With all three systems shown in Fig. 2, we carried out a SWV frequency scan from 3–1000 Hz, and the resultant data was used to determine the electrochemical critical times with the method describe by Komorsky-Lovrić and Lovrić [62]. Specifically, plotting peak current over frequency (*I*_peak_/*f*_SWV_) versus inverse frequency (1/*f*_SWV_) or time, this method can give useful information about the total amount of charge transferred at various measurement times. The peaks in such a plot are referred to as the “electrochemical critical times” under varying conditions. In our system, these critical times represent a convolution of both tethered diffusion of the MB label and electron transfer from MB to the electrode—effects that cannot be separated. Nonetheless, the critical times determined with this approach are very useful in evaluating the overall assay mechanistically.

**Fig. 3:**
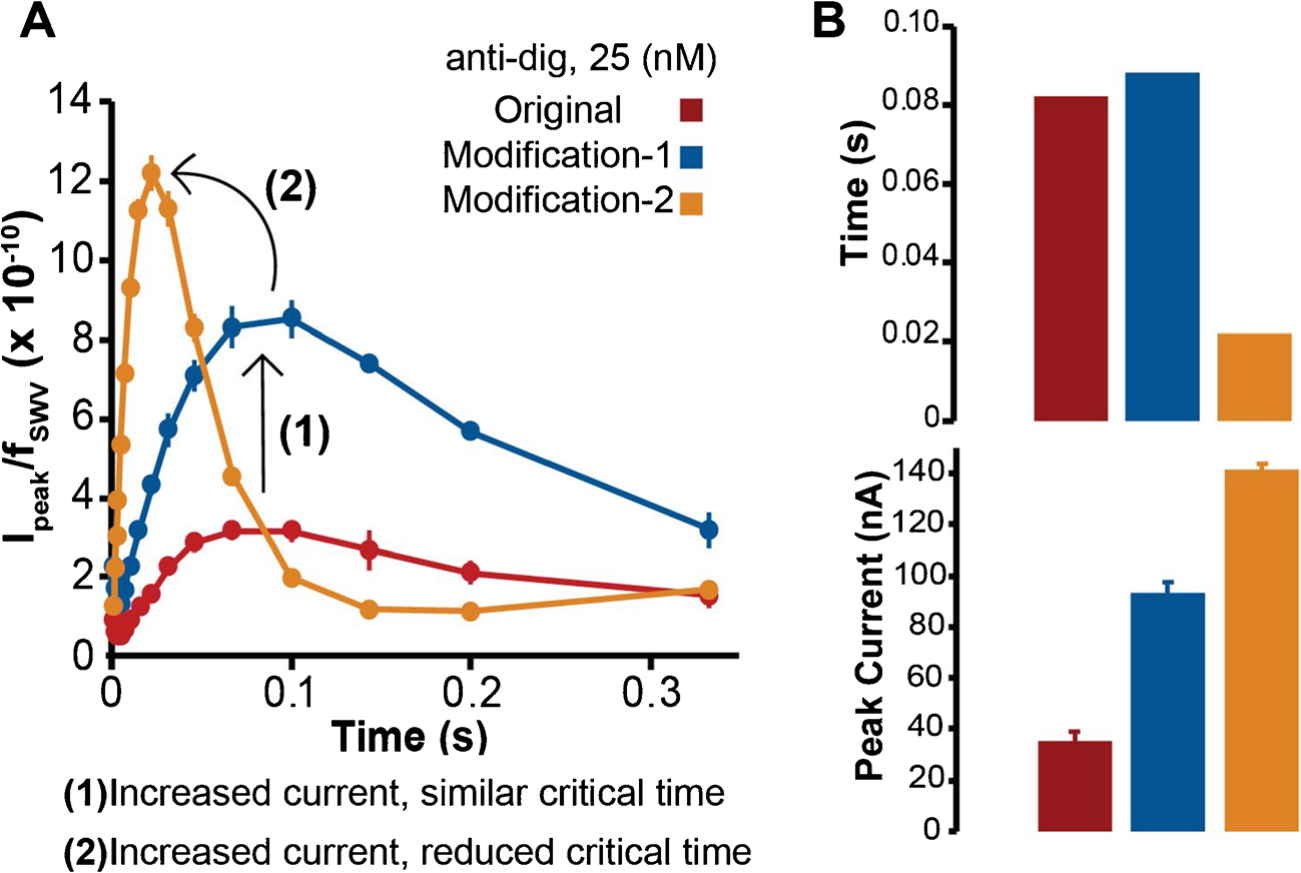
Analysis of electrochemical critical times with all three systems gave mechanistic insights into the positional effects of the flexible linkers. (**A**) Compared to the original system (red), an enhancement to DNA hybridization from modification-1 (blue) gave more current with equivalent critical time, labeled by arrow (1) in part A. Modification-2 (orange) gave more current and a faster critical time as a result of the increased tethered diffusion rate, labeled by arrow (2) in part A. (**B**) Magnitude comparisons of electrochemical critical times and peak currents

As determined by the data in Fig. 3A, the **original** system exhibited a critical time of 82 ms (red), and the **modification-1** system gave an obvious increase in current with an essentially equivalent critical time of 88 ms (blue). This can be explained since the site of this flexible linker modification was positioned away from the electrode and above the MB label; thus, it should not significantly alter the tethered diffusion of the MB label near the surface. The positioning of the MB label is controlled mainly by the 7-bp dsDNA segment, which is positioned below the linker. This analysis further supports our assertion that **modification-1** mainly results in improved MB-DNA and thio-DNA hybridization energy, causing the recruitment of more antibody-antigen-DNA complexes to the surface. Arrow (1) in Fig. 3A shows that the overall effect of **modification-1** was an increase in current without a change to the critical time.

By contrast, Fig. 3A shows that **modification-2** (orange) resulted in a significant enhancement in both the current and the electrochemical critical time, as labeled by arrow (2). Following the tethered diffusion model of Huang and White [63], the more flexible tether to the electrode surface appears to have caused a significant increase in the tethered diffusion rate of the MB label, resulting in more interactions of MB with the electrode over time, thereby giving more current. The data shows the critical time in **modification-2** to be 22 ms, about four times faster than the other systems.

The effects of flexible spacer position on both critical time and peak current are also shown in Fig. 3B. Overall, these results further confirmed our previous findings that introducing sufficient probe flexibility into a multi-strand DNA-based proximity assay leads to improved assay performance [53].

### Effect of ionic strength on assay performance

**Fig. 4:**
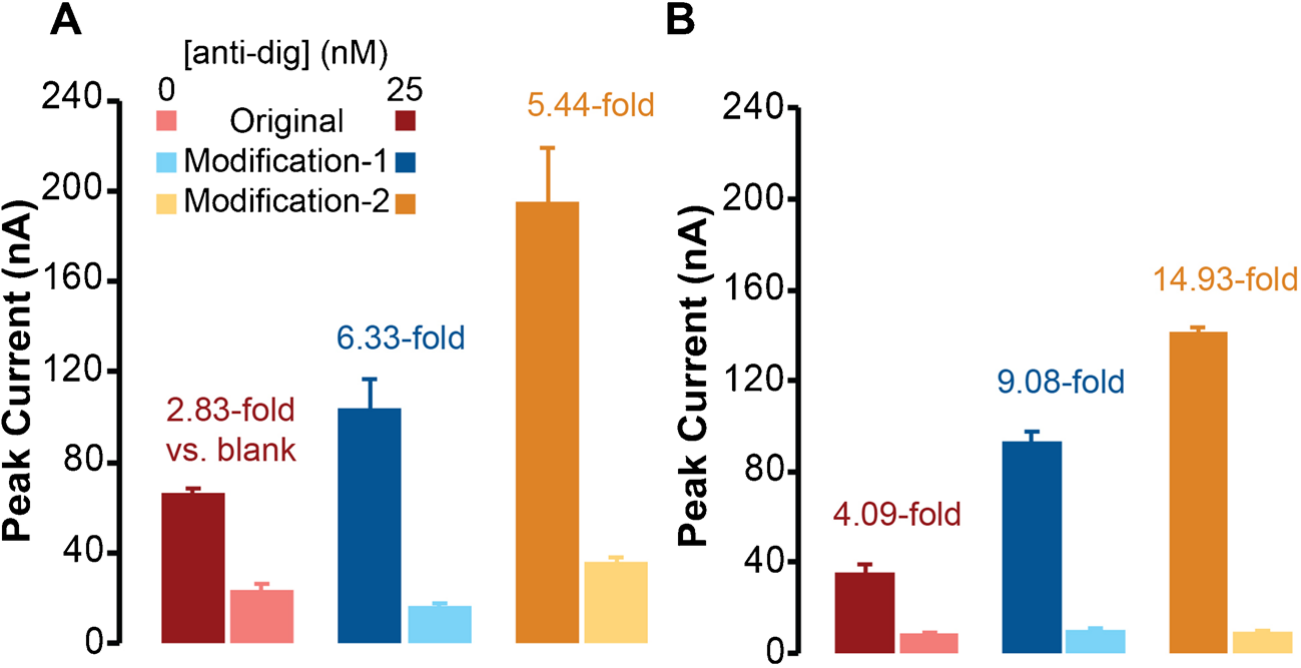
Peak current dependence on salt concentration for all three systems. **A** Currents increased in 1.0 M NaCl for all systems with both signal (25 nM anti-dig) and background (0 nM anti-dig) compared to (**B**) experiments with 0.5 M NaCl in buffer. This effect is attributed to enhanced DNA hybridization with increased salt. The fold increases of signals compared to the blanks are shown above each set of measurements

It is well known that increased ionic strength can stabilize DNA hybridization [64]. Recently, we have shown that increasing salt concentrations in measurement buffer can also reduce the thickness of the electric double layer, allowing more efficient hybridization of short DNA strands near the electrode surface [61]. Therefore, with the objective of improving assay performance further, we carried out experiments for all three design systems with 1.0 M NaCl in the buffer and compared those results to the 0.5 M NaCl results from earlier studies. Figure 4 shows that target-dependent peak currents were improved with increased salt concentration, while we also observed an increase in BG currents. Since we had already introduced spacers into all designs in this work, we expect that the electric double-layer effects on DNA hybridization [61] were minimized. Thus, the increased currents observed in 1.0 M NaCl were likely a result of enhanced DNA hybridization, which would affect both signal and background complexes as observed. Although signal currents were improved, the 1.0 M salt also enhanced background; thus, the 0.5 M salt system was chosen as optimal for sensor calibration.

### Sensor calibration

**Fig. 5:**
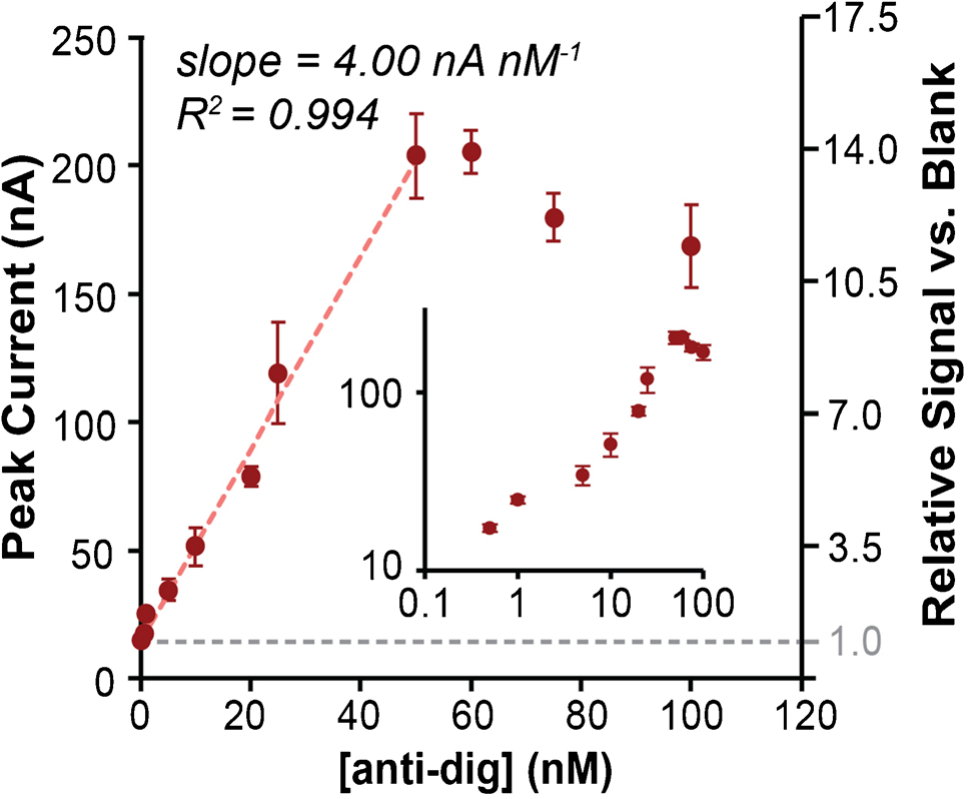
Calibration curve for anti-digoxigenin antibody sensing. For the optimized system using modification-2, a sensitivity of 4.0 nA nM^−1^, an LOD of 300 pM, and a dynamic range of two orders of magnitude (0.5 to 50 nM) were observed by using either maximum (peak) current or signal-to-background ratio. Inset shows a log–log plot of the same data to enhance the view of the dynamic range. Error bars represent standard deviation of triplicate measurements with three different electrodes

Next, the optimized sensor’s response toward varying antibody concentrations was evaluated. Here, the **modification-2** system was characterized with 0.5 M NaCl in the buffer. As shown in Fig. 5, the system responded to anti-dig addition as expected with a sensitivity of 4.0 nA nM^−1^ and a 3*σ* LOD of 300 pM, with a dynamic range up to 50 nM. As in all proximity assays [13, 48], at high analyte concentration, once the probes become the limiting reagent, signal was shown to decrease. If desired, increases in the dynamic range should be achievable by increasing the amounts of antigen-DNA strands. It would also be interesting to study the effects of thio-DNA surface coverage density on the current and its relation to probe flexibility. Finally, since adding both flexible linkers into the system gave signal enhancements without background enhancements (see Fig. 2), we depicted the percent change in current in the secondary *y*-axis in Fig. 5. Inclusion of the flexible spacers gave a sensor that exhibited approximately an increase in current upon addition of 50 nM antibody that was 14-fold higher than the blank.

### Anti-digoxigenin sensing in human serum

**Fig. 6:**
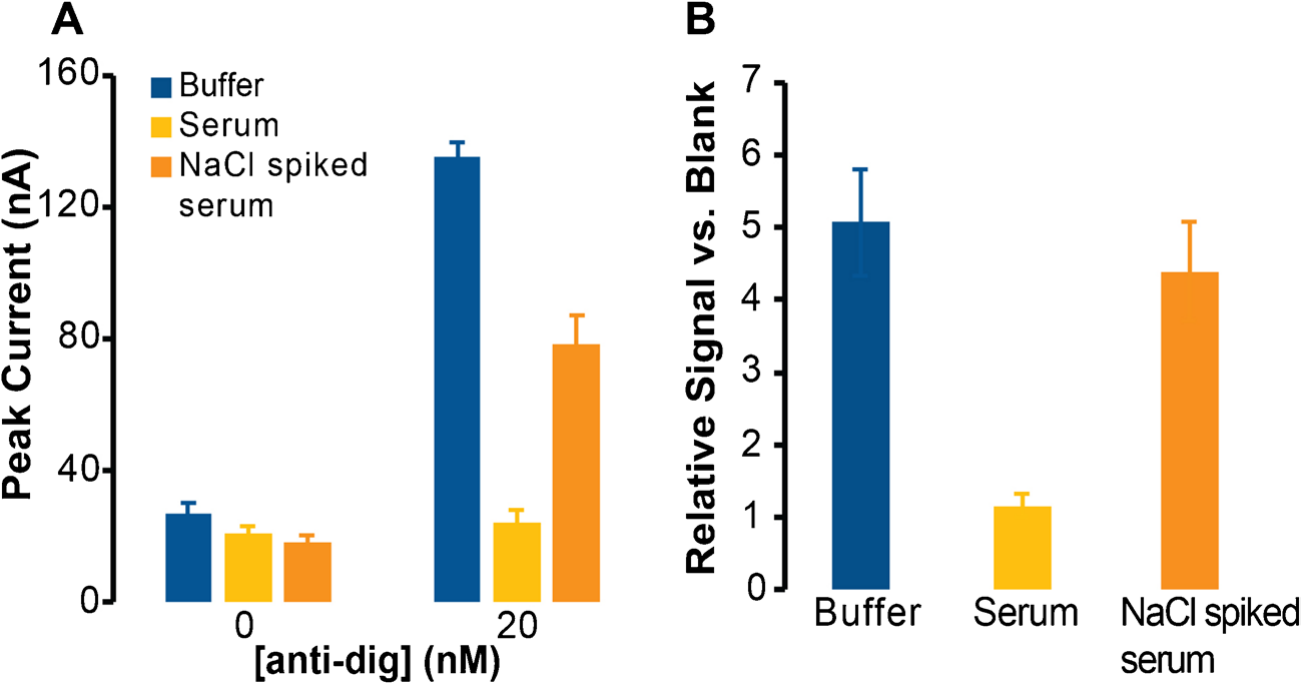
Assay functionality in 90% human serum. (**A**) Raw peak currents showing changes for 20 nM anti-digoxigenin spiked in serum and in serum spiked with NaCl. The raw currents were lower for salt-spiked serum compared to the buffer control. (**B**) However, the relative signal (versus blank) demonstrated comparable results to that of the buffer control. The results also show that spiking a high concentration of salt can improve this assay’s performance in serum. Error bars report standard deviations from three separately fabricated sensors

To test the functionality of the sensor in biological fluids as well as the selectivity, we spiked 20 nM of anti-dig into human serum (90%). Initially, in comparison to the buffer control, we did not observe a significant increase in signal, compared to the BG. Because serum has approximately 135–145 mM of salt [65], and since we have shown salt concentration to play an important role in hybridization of short DNA strands to surfaces in EC sensors [61], we hypothesized that this comparatively low salt concentration in serum may prevent target-dependent DNA hybridization, in combination with other factors such as serum interferents. Therefore, a small volume of higher concentration of salt (4 M) was spiked to increase the final concentration of salt in the serum samples to around 0.45 M (avoiding too much dilution), near the optimal concentration of 0.5 M reported in Fig. 4. This change enabled observable, antibody-dependent currents to be measured, although the raw current for 20 nM anti-digoxigenin was lower than that of the buffer control (Fig. 6A). However, considering the likelihood of serum interferences blocking some of the surface, as well as sensor-to-sensor variabilities, we also evaluated the percentage change in current. Using the percentage method, the NaCl spiked serum showed results comparable to that of the buffer control, within error (Fig. 6B). We attribute these differences in raw peak currents to either electrode-to-electrode variability or to recently reported challenges in serum or blood measurements, including biofouling, voltage-induced desorption, or competitive displacement by serum thiols [66, 67]. Even with these interferences present, our signal-on sensors were able to consistently record a response to antibodies in 90% human serum, and the relative change in signal was nearly equivalent to that in buffer.

### Assay limitations

With any new assay development, it is important to note its limitations for future use. First, although this work used commercially synthesized modified oligos, the synthesis of analyte-DNA conjugates (such as dig-DNA) for future iterations of this antibody-sensing ECPA will require custom synthesis and purification. Control over the stoichiometry and yield of these reactions can sometimes be problematic. This could be costly unless a standard synthetic protocol is developed on site for each targeted analyte. Furthermore, the existing workflow for this assay requires about 71 min, although this is addressable, and future work will seek to reduce the timing and the number of steps.

Another potential issue is that antibodies may no longer bind to analyte-DNA conjugates if the structure of the conjugate differs significantly from the hapten used to select the antibody. In these cases, the best solution is trial and error, and multiple antibodies may need to be screened.

A cost analysis for the presented version of the assay has been provided as Table S-2 in the Supporting Information. Interestingly, while the PEG spacer modifications did increase the assay and calibration costs slightly, the bulk of the assay cost came from the fabrication of the electrode and PDMS wells.

## Conclusions

In this work, we have extended the previously developed ECPA sensing approach for antibody detection. Similar to our recent work leveraging TFA [53], we have again shown the importance of probe flexibility in improving the performance of this electrochemical proximity-based assay. Incorporating PEG-modified MB-DNA and thio-DNA strands gave a drastic improvement in target-dependent current and permitted an LOD of only 300 pM using a direct EC measurement (no signal amplification). Although we developed this sensor for anti-digoxigenin antibody detection as a proof-of-concept, we expect that the sensor could be modified to detect more clinically relevant antibodies in the future, particularly in cases where generated antibodies build into the upper pM or nM concentration ranges. Overall, with a 30-min antibody/dig-DNA incubation, a 40-min electrode incubation, and an approximately 1-min measurement (at a single SWV frequency), the assay workflow currently requires about 71 min. Future iterations of the method will explore mixing of all reagents and measurement in one solution to reduce workflow time, as shown feasible with our reusable ECPA method [50]. It is expected that autoimmune disease monitoring, in particular, could benefit greatly from such a technology.

Along with improving performance, the flexible linkers also revealed several novel and interesting mechanistic details of ECPA, augmented by varying SWV frequency and studying the electrochemical critical times. By adding flexibility away from the electrode (**modification-1**), we could increase current without a change to the critical time, while addition of flexibility to the electrode tether strand (**modification-2**) gave enhancements to both current and electrochemical critical time. These results were explained by the differential effects of flexibility on either DNA hybridization energy or tethered diffusion rate of the EC label. These changes gave large increases in current to the ECPA sensor, and we expect that our method to improve surface-tethered flexibility could be applied to enhance current in other DNA-driven electrochemical biosensors [46], such as aptamer-based sensors.

## Supplementary Information

Below is the link to the electronic supplementary material.

Supplementary file1 Supp_info_ECPA_final_ESM.pdf (PDF file): This document includes a list of DNA sequences used, a sensor cost analysis, electrode fabrication details, data analysis demonstrations, and calibrations for the three sensor system designs. (PDF 977 KB)

## Declarations

### Source of biological material

Human serum samples were purchased from a specimen supplier and were de-identified and prescreened for pathogens.
