# Supplementary material for: An electrochemical proximity assay (ECPA) for antibody detection incorporating flexible spacers for improved performance
Source: Anal Bioanal Chem. 2024 Oct 5;416(28):6529–39. doi: 10.1007/s00216-024-05546-9 (PMC11541272; doi:10.1007/s00216-024-05546-9)
Supplement: Supplementary file 1 — Supplementary file1 Supp_info_ECPA_final_ESM.pdf (PDF file): This document includes a list of DNA sequences used, a sensor cost analysis, electrode fabrication details, data analysis demonstrations, and calibrations for the three sensor system designs. (PDF 977 KB) [file 216_2024_5546_MOESM1_ESM.pdf]

Supporting Information for:

An Electrochemical Proximity Assay (ECPA) for Antibody Detection Incorporating Flexible Spacers for Improved Performance

*Amanda S. N. Kurian, Mainul Islam Mazumder, Asanka Gurukandure, and Christopher J. Easley**

Department of Chemistry and Biochemistry, Auburn University, Auburn, AL, 36849

Email: chris.easley@auburn.edu

Supporting Information (SI) Contents:

Page S-2: Sequences of DNA strands used in this study, **Table S-1.**

Page S-3: Cost analysis of the presented assay, with and without PEG spacers, **Table S-2.**

Page S-4: Electrode and cell fabrication details; surface density calculations,

Page S-5: Photomask, gold-on-glass electrodes, and 3D-printed well template, **Figure S-1.**

Page S-6: Data analysis (MATLAB) with minimal nonfaradaic background, **Figure S-2.**

Page S-7: Data analysis (MATLAB) with significant nonfaradaic background, **Figure S-3.**

Page S-8: Comparisons of calibration curves for systems using the original system, the modification-1 system, and the modification-2 system, **Figure S-4.**

Sequence name	Sequence (5' to 3')
**Digoxigenin tagged DNA (dig-DNA)**	TAG GAA AAG GAG GAG GGT GGC CCA CTT AAA CCT CAA TCC A/3DiG_N/<br><br>20 underlined nucleotides are complimentary to those in MB-DNA or PEG-MB-DNA below.
**Thiolated DNA (thio-DNA)**	/5ThioMC6-D/AAA AGC ATG GTA TTT TTC GTT CGT TAG GGT TCA AAT CCG CG<br><br>7 green, underlined nucleotides were complimentary to those in MB-DNA or PEG-MB-DNA below.
**PEG modified thiolated DNA (PEG-thio-DNA) "modification-2"**	/5ThioMC6-D/iSp18/GCA TGG TAT TTT TCG TTC GTT AGG GTT CAA ATC CGC G<br><br>7 green, underlined nucleotides were complimentary to those in MB-DNA or PEG-MB-DNA below.<br>1 spacer (iSp18) equivalent length = 2.4 nm = ~4 nt
**Methylene blue DNA (MB-DNA)**	CCA CCC TCC TCC TTT TCC T AT CTC TCC CTC GUC ACC AUG C /MB-C7/<br><br>11 red nucleotides were replaced by spacers below.<br>20 underlined nucleotides are complimentary to those in dig-DNA above.
**PEG modified methylene blue DNA (PEG-MB-DNA) "modification-1" and "modification-2"**	CCA CCC TCC TCC TTT TCC T /iSp18//iSp18//iSp18//iSp18/ GTC ACC ATG C [AmC6-Glen][MB-Bios]<br><br>7 green, underlined nucleotides were complimentary to those in thio-DNA or PEG-thio-DNA above.<br>4 spacers equivalent length = 9.6 nm = ~14 nt

Table S-1. DNA sequences used in this study. dig-DNA, thio-DNA, and PEG-thio-DNA were purchased from IDT. MB-DNA was obtained from Biosearch Technologies. PEG-MB-DNA was purchased from Biosynthesis. Complimentary regions are signified by underlined and/or green colored text. Spacers are signified by red text.

Item	18-electrode cost (USD)	1-electrode cost (USD)
**Thio-DNA, no PEG**	$0.246	$0.014
**Thio-DNA, with PEG**	$0.643	$0.036
**MB-DNA, no PEG**	$0.270	$0.015
**MB-DNA, with PEG**	$1.130	$0.063
**Dig-DNA**	$0.841	$0.047
**Anti-dig**	$2.850	$0.158
**GoG**	$8.580	$0.477
**AZ 40XT photoresist**	$4.200	$0.233
**AZ 300 MIF developer**	$2.250	$0.125
**PDMS**	$5.47	$0.304

		Cost (USD)	Added Cost (USD)
**Original Assay (no PEG)**	1 sample, triplicate runs	$3.64	-
	6-point calib. Curve, triplicate runs	$24.71	-
**Modification-1**	1 sample, triplicate runs	$3.79	**$0.14**
	6-point calib. Curve, triplicate runs	$25.57	**$0.86**
**Modification-2**	1 sample, triplicate runs	$3.85	**$0.21**
	6-point calib. Curve, triplicate runs	$25.97	**$1.26**

Table S-2. Cost analysis of the presented assay, with and without PEG spacers. The cost of each component for a full 18-electrode experiment (see Figure S-1) is compared alongside a single-electrode experiment (top). The costs of the three variations of the assay reported in the main text (Original, Modification-1, Modification-2) are also reported (bottom), and the added costs of the PEG spacer modifications are reported at the bottom right. The final version only increased the cost by $0.21 for a triplicate sample run and $1.26 for a full calibration curve.

Notes: Antibody costs were excluded from triplicate sample runs, and buffer costs were considered negligible. Units are reported in US dollars (USD) rounded to the nearest tenth of a cent.

Gold electrode fabrication

A standard photolithographic procedure was followed for gold electrode fabrication, using the positive photoresist, AZ40XT. A photomask of the electrode was designed in Adobe Illustrator and was sent to Fine Line Imaging (Colorado Springs, CO), where a positive photomask was printed (Figure S1-A). Based on this design, 18 electrodes of 2 mm diameter can be fabricated on a gold-on-glass (GoG) slide. After patterning the gold slide, it was dipped in gold etchant (30 s), followed by chromium etchant (15 s) to remove the unexposed gold and chromium. The AZ photoresist was removed by immersing slides in DMSO for 30 min at 110-150 °C. The etched electrodes were then washed with deionized water and dried with nitrogen.

Electrochemical cell fabrication

The electrochemical (EC) cell was fabricated using polydimethylsilane (PDMS), followed by bonding with fabricated GoG electrode. A 3D CAD file was designed in either Sketchup or Solidworks and the designed mold was printed using the Makerbot Replicator 2 printer, using polylactic acid (PLA) filament (1.75 mm diameter) (Figure S1-B). PDMS and curing agent were mixed in a ratio of 10:1 and poured on to a silicon wafer. The printed 3D mold was then placed on this wafer carefully, followed by baking the PDMS for 1 hr at 60°C. After 1hr, the PDMS was removed from the oven and carefully separated from the mold and wafer. The PDMS wells are then placed in an oven at 100°C to complete curing any remaining curing agent, for at least 2-3 hrs. When the GoG slides are ready to be used, the PDMS is cut into the desired number of wells, depending on the number of electrodes to be used. The etched GoG is cut into the desired number of electrodes and first cleaned with piranha solution (H_2SO_4 : H_2O_2, 3:1) for 1 min. The electrodes are then washed with deionized water and dried with nitrogen. Next, the cut PDMS and cleaned electrodes are sonicated in methanol, followed by drying with nitrogen. The cleaned PDMS and GoG electrodes were then attached to each other by plasma oxidation, to finally form the EC cell (Fig S1-C). Each PDMS well-bound GoG can carry ~100 μL of solution.

Surface density calculation

The number of moles of hybridized MB-DNA, N_{tot}, was determined using the following equation,

$$i_p = 2nfFN_{tot} \times \frac{sinh\left(\frac{nFE}{RT}\right)}{cosh\left(\frac{nFE}{RT}\right) + 1}$$

where i_p is the peak height of the current measurement, n is the number of electrons transferred per redox event (with MB label, n = 2), F is the Faraday constant, R is the universal gas constant, T is the temperature, E is the applied voltage amplitude, and f is the SWV frequency. The number of moles of MB-DNA was divided by the electrode surface area to obtain the surface density in units of mol cm^{-2}. For the **original** design, **modification-1**, and **modification-2**, the surface densities were 1.14, 1.45, and 1.30 fmol cm^{-2}. Since these values were similar and showed no obvious trends, we assumed that the flexible spacers did not have an appreciable effect on the surface packing density of the thiol-DNA strands.

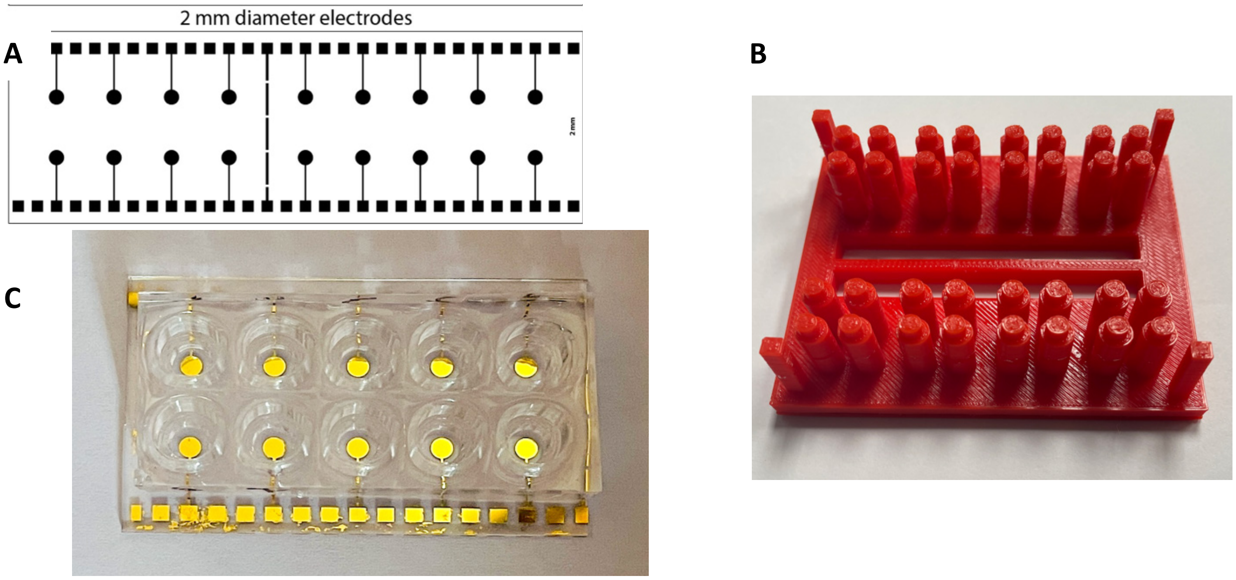

Figure S-1. (A) Adobe illustrator design of the positive photomask showing 18, 2-mm diameter electrodes. (B) A 3D mold used to fabricate PDMS wells. Figure shows a 32-well mold. (C) An EC cell after plasma oxidation and bonding of the glass to the PDMS. The figure shows 10 independent GoG electrodes ready for thiolated DNA immobilization.

Data Analysis

After data acquisition, a customized Windows batch file was first used to convert batches of multiple SWV data files (.dta) acquired with the Gamry 600 potentiostat into text files (.txt) for further analysis. Next, a customized MATLAB code was used to extract the data from the converted text files. Figure S-2 below is an example which shows the step-by-step data extraction for obtaining peak currents at 464 Hz; here, we detected 25 nM anti-dig using the modification-2 system. The code extracts and separates the baseline (nonfaradaic) current from the Faradaic current using a third-order polynomial fit to the baseline; this is done for forward, reverse, and difference currents. For any other SWV frequencies used, a similar method was followed.

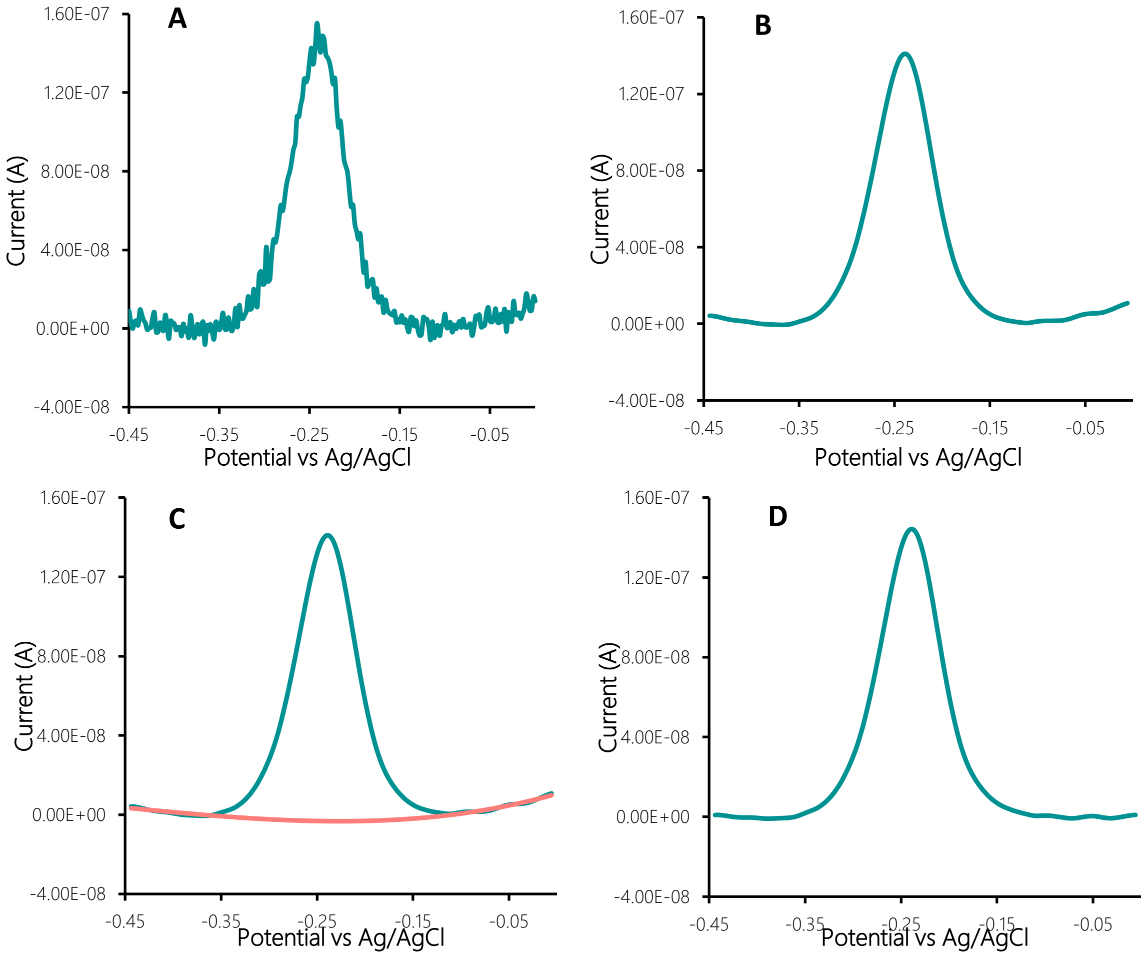

Figure S-2. A step-by-step depiction of analysis of data to separate faradaic from nonfaradaic currents using customized MATLAB code; this example uses data with *minimal* nonfaradaic background. (**A**) Raw currents. (**B**) A plot showing the smoothed raw data from (A), where a 21-point smoothing filter was used. (**C**) Baseline correction, using a 3rd order polynomial fit (peach curve shows the baseline fit). (**D**) The baseline corrected faradaic current. Peak currents for all measurements were obtained from the maximum current of this type of faradaic curve.

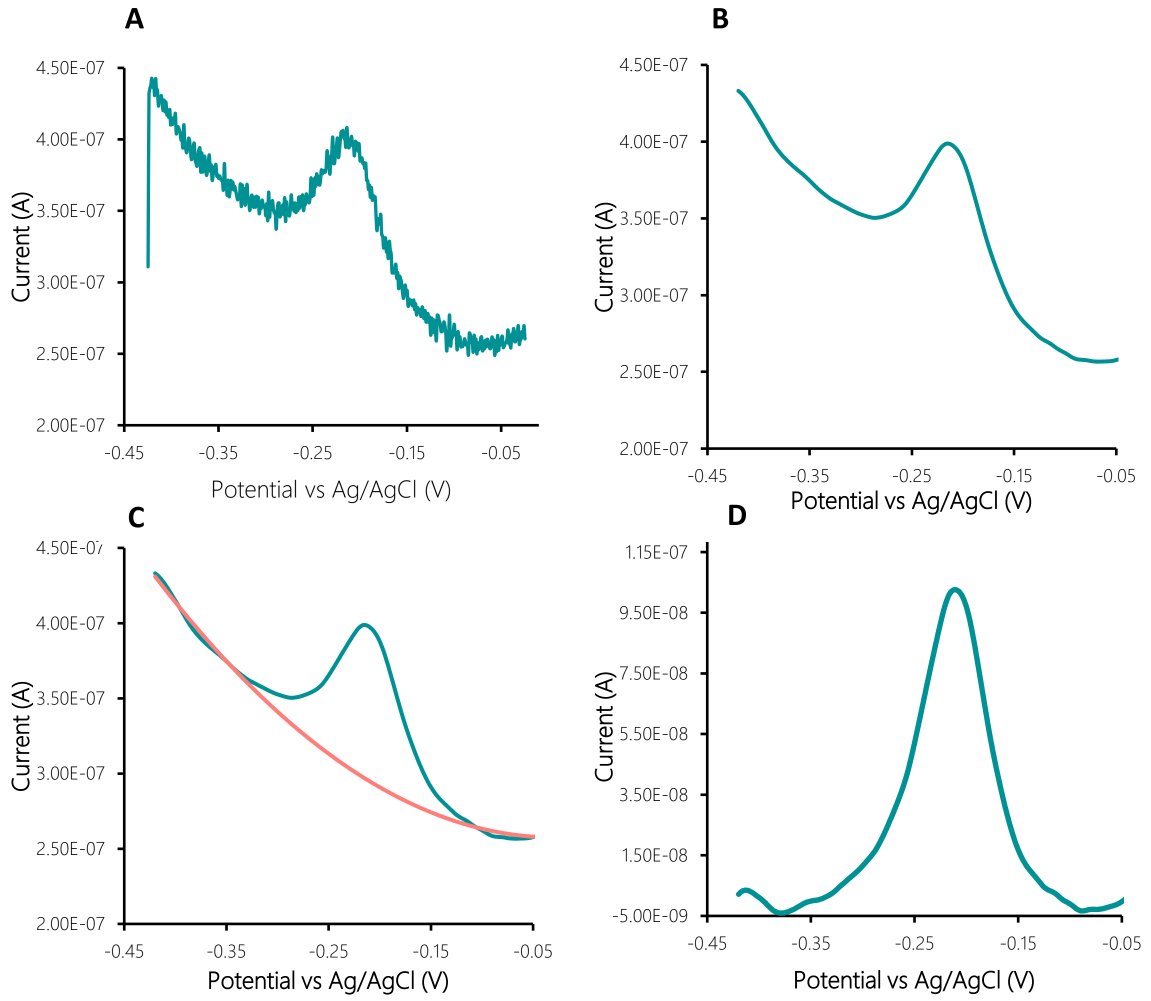

Figure S-3. A step-by-step depiction of analysis of data to separate faradaic from nonfaradaic currents using customized MATLAB code; this example uses data with *significant* nonfaradaic background. (**A**) Raw currents. (**B**) A plot showing the smoothed raw data from (A), where a 21-point smoothing filter was used. (**C**) Baseline correction, using a 3rd order polynomial fit (peach curve shows the baseline fit). (**D**) The baseline corrected faradaic current. Peak currents for all measurements were obtained from the maximum current of this type of faradaic curve.

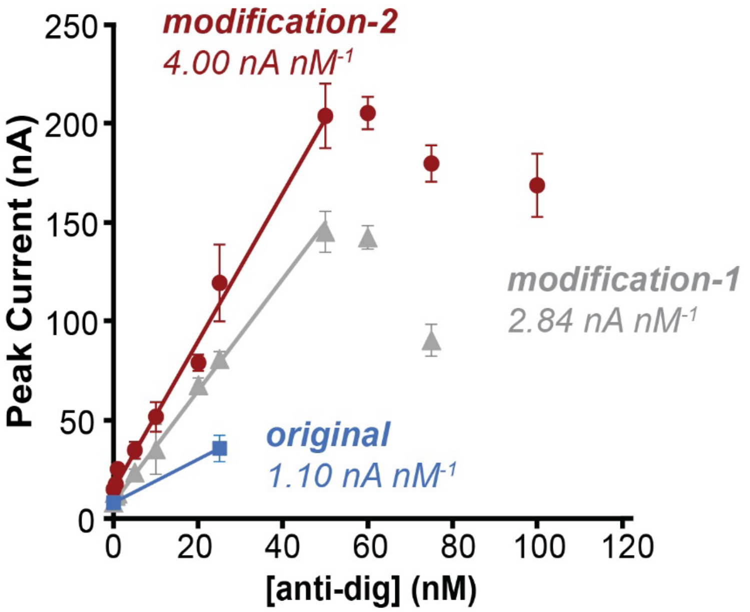

Figure S-4. Comparisons of calibration curves with the three systems. As shown in the figure, the **modification-2** system exhibited a 1.4-fold improvement on the **modification-1** system and a 3.6-fold improvement on the **original system**. These data further confirm the benefits of the flexible linkers.
